# The U.S. Transgender Survey of 2015 Supports Rapid-Onset Gender Dysphoria: Revisiting the “Age of Realization and Disclosure of Gender Identity Among Transgender Adults”

**DOI:** 10.1007/s10508-023-02754-9

**Published:** 2023-12-18

**Authors:** Leor Sapir, Lisa Littman, Michael Biggs

**Affiliations:** 1 Manhattan Institute for Policy Research, 52 Vanderbilt Ave., New York, NY 10017 USA; 2 The Institute for Comprehensive Gender Dysphoria Research, Providence, RI USA; 3 Department of Sociology, University of Oxford, Oxford, UK

## Introduction

“Rapid-onset gender dysphoria” (ROGD) describes a presentation in a recent cohort of adolescent and young adults who first became gender dysphoric or trans-identified during or after the onset of puberty (Littman, 2018, 2021). The ROGD hypotheses are, briefly stated, that this relatively new and distinct clinical presentation of late-onset gender dysphoria exists, and that psychosocial factors, including social influences (social media, social and peer contagion, etc.), maladaptive coping mechanisms, mental health conditions, and other stressors can contribute to its appearance in some individuals (Littman, 2018, 2021).

In “Age of Realization and Disclosure of Gender Identity Among Transgender Adults,” Turban et al. (2023a) claim to find evidence against ROGD. Relying on data from the U.S. Transgender Survey of 2015 (USTS-15) (James et al., 2016), Turban et al. divided respondents into two groups—early realization and late realization—based on whether they “realized their TGD [transgender and/or gender diverse] identities” before or after age 10. They found that 59.2% of respondents had early realization, and that the median time from realization to disclosure of their identities to others was 14 years. Thus, Turban et al. conclude, “it is likely that gender dysphoria experienced by many…TGD youth is not ‘rapid-onset,’ but rather that TGD youth disclose their TGD identities to their parents and others years after their personal realization.”

We write to point out problems with their analysis. Turban et al. (1) misstate the ROGD hypothesis, (2) analyze the wrong age cohorts in USTS-15, (3) use a dubious proxy for “realization,” (4) use an unreasonable definition of “disclosure,” (5) provide misleading analysis of time to disclosure, (6) misrepresent and underestimate the significance of their sample’s female skew, and (7) omit ROGD-relevant data pertaining to respondents’ mental health. When these flaws are acknowledged and the data are accurately reported, the USTS-15 actually provides support for the ROGD hypothesis.

## Misstatement of Rapid-Onset Gender Dysphoria Hypothesis

Turban et al. claim that the theory of ROGD assumes that “[transgender and gender diverse] identities associated with ‘later realization’ are transient and will not continue into adulthood.” However, transience was never theorized to be inherent to ROGD. The stability of ROGD remains an urgent question for longitudinal research. Nevertheless, even or especially if ROGD-related identity is transient, the USTS-15 is ill-equipped to test identity duration because only those who currently identified as “transgender, trans, genderqueer, and non-binary” were allowed to participate. Those who once identified as TGD but no longer did so by 2015 were precluded from taking the survey. Specifically, desisters and detransitioners, the group that would be most associated with “transient” transgender identity, would have been excluded from participating in the USTS-15. By Turban et al.’s own understanding of ROGD, therefore, their conclusions are highly dubious due to reliance on a sample with severe selection bias.

Oddly, two of the authors (Turban and Keuroghlian) have acknowledged this exact problem of selection bias in a previous article that relied on USTS-15 to examine “factors leading to detransition” (Turban et al., 2021). As Turban et al. wrote in the limitations section of that article:

The generalizability of our study is limited by the nonprobability sampling design of the USTS…Because the USTS only surveyed currently TGD-identified people, our study does not offer insights into reasons for detransition in previously TGD-identified people who currently identify as cisgender.

A survey that does not include “previously TGD-identified people who currently identify as cisgender” cannot credibly test for the transience of transgender identity.

## Wrong Age Cohorts

The USTS-15 is a survey of adults of all ages. Turban et al. muddy the waters, however, by lumping together USTS-15 respondents who were and those who were not within the timeframe relevant to ROGD. As we discuss below, this method enables Turban et al. to conceal ROGD-relevant information.

The ROGD hypothesis pertains to recent trends, not to people who were in their 40s in 2015. Social media is one of the proposed contributors to ROGD (Littman, 2018, 2021) and the use of social media only took off in the late 2000s. Moreover, data from a number of Western countries have shown that the rates of gender dysphoric adolescents seen in gender clinics rose rapidly from the late 2000s to the late 2010s (Aitken et al., 2015; de Graaf et al., 2018; Zhang et al., 2021). This means that only the 18–24 year-old age group of the USTS-15 could have had experiences recently enough to explore the ROGD hypotheses. The older participants are not part of the ROGD-relevant age cohort.

To explore whether individuals who fit the ROGD presentation (“late realization”) had a short time frame from realization to disclosure, Turban et al. could have analyzed, in the ROGD-relevant age cohort, just the “late realization” group. Alternatively, they could have analyzed both the late and the early realization groups within this age cohort and compared them. Instead, Turban et al. analyzed only the early realization group for the entire sample combined.

## Dubious Proxy for “Realization”

Turban et al. conceptualize time to disclosure as the length of time between the age respondent “first came to realize their TGD identities” and the age at which they “first start[ed] to tell others they [were] trans (even if [they] did not use that word).” However, USTS-15 did not ask respondents about when they “first came to realize their TGD identities.” The three relevant questions that appear on the survey are:

**3.1 **At about what age did you begin to feel that your gender was “different” from your assigned birth sex?

**3.2 **At about what age did you start to think you were trans (even if you did not know the word for it)?

**3.3 **At about what age did you first start to tell others that you were trans (even if you did not use that word)? [Or] I have not told others that I am trans.

Turban et al. interpret answers to Question 3.1 (Q3.1) as the age at which respondents “first came to realize their TGD identities.” By combining transgender and gender diverse into a single category (“TGD identity”), despite these being distinct (if overlapping) experiences, Turban et al. are able to equate “start to feel your gender was ‘different’ from your assigned birth sex” with “start to think you were trans,” and “start to think you were trans” with “first came to realized their TGD identities.”

We question this interpretive sequence. First, the word “gender” in Q3.1 is ambiguous. “Gender” can be used a synonym for sex but it can also refer to the social and psychological beliefs and expectations associated with sex. “Feeling that your gender is different from your assigned birth sex” can therefore mean feeling you are the wrong sex, but it can also mean feeling you don’t conform to beliefs and expectations associated with your sex—in other words, gender nonconformity. ROGD is about the onset of gender dysphoria in adolescents with no history of gender identity problems and/or gender dysphoria. An adolescent who was merely gender nonconforming as a child can therefore experience ROGD, according to the original hypothesis. Second, ROGD is hypothesized to arise in the context of a global movement to make visible and normalize transgender identity. Thus, ROGD posits a close connection between the appearance of gender dysphoric feelings in a subset of adolescents and young adults and their exposure to and adoption of a transgender identity as a way of making sense of those feelings.

Given these key background facts, Q3.2 is the more natural option for assessing “age of realization of TGD identities” in USTS-15 because it asks about the acquisition of a transgender identity explicitly. At minimum, Turban et al. should have explained why they chose their counterintuitive approach.

**Fig. 1:**
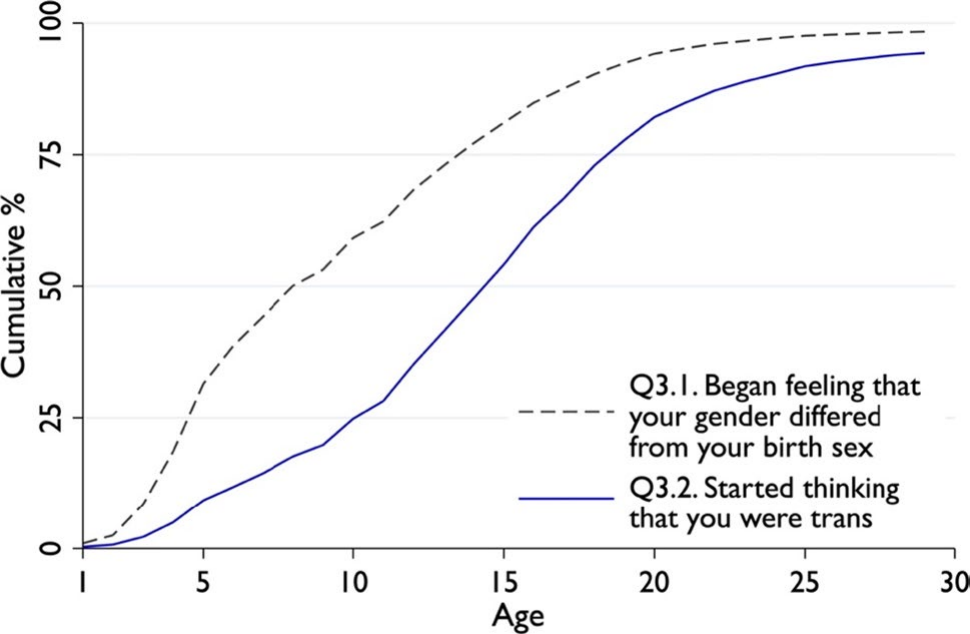
Age of realization in USTS according to two different questions

Turban et al. withhold critical information about the discrepancies between age of first gender incongruent feelings (Q3.1) and age at which respondents “start[ed] to think [they] were trans” (Q3.2) in the USTS-15. Using the entire USTS-15 sample, Fig. 1 shows the significant difference between these two questions. Respondents reported feeling that their gender differed from their natal sex at a median of 8 years of age. This is what Turban et al. described as the “age of realization.” Yet respondents recalled thinking that they might be trans only at a much older age: the median was 15 years.

Why did Turban et al. rely on Q3.1 when Q3.2 is the more natural proxy for “first realizing a TGD identity”? A possible reason is that using Q3.2 would result in a finding that late realization (75%) was three times more common in the entire USTS-15 sample than early realization (25%), and not 40.8% as reported by Turban et al. In the ROGD-relevant age cohort (18–24), the breakdown (using Q3.2) is 83% late realization compared to 17% early realization. Although Turban et al.’s results support the ROGD hypothesis, as they document that participants with late realizations exist, using Q3.2 would have shown that the ROGD presentation was quite common. Using Q3.1 allowed Turban et al. to assert a much higher incidence of early realization in the USTS-15 than is supported by the relevant data. This, in turn, allowed them to report a longer time from realization to disclosure.

## Unreasonable Definition of “Disclosure”

Turban et al. make clear that a main purpose of their article is to challenge the use parental reports of identity disclosure (Littman, 2018) as unreliable for assessing the timing of transgender identification onset. As they wrote, “assertions based solely on data from persons who are not privy to internal identity processes among TGD youth, such as the parental respondents whose data the ROGD hypothesis was principally derived from, may be largely inaccurate.”

We think Turban et al. have created a straw-man. The theory of ROGD does not assume that parents alone possess reliable knowledge about their children’s identity. In their effort to rebut this straw man, Turban et al. overcorrected to the opposite extreme. In their view, “internal identity processes,” as reported retrospectively by adults recruited for a survey through transgender advocacy organizations, are alone enough to determine when an individual’s “TGD identity” first emerged. Since Turban et al.’s reasoning has appeared in the literature before (Bauer et al., 2022), we think it is necessary to clarify that both “internal processes” (which are more subjective) and parental reports (which are more objective) are relevant to the question of identity development and disclosure.

USTS-15 did not survey the parents or legal guardians of TGD respondents and thus contains no information about when the parents/guardians first noticed gender non-conforming behaviors in their children. Turban et al. appear to assume that, but for their child’s explicit disclosure of identity, parents or guardians would have been in the dark about their child’s gender experiences. This assumption contradicts decades of research and clinical experience. In a common gender clinic scenario, parents observe gender atypical behavior and verbalized cross-gender desires in their child at a very young age, take their child to see a gender specialist, and receive a diagnosis of gender dysphoria. If the child is young enough, he or she may not even fully comprehend the true nature of the clinical interaction. It strikes us as highly implausible that parents of USTS-15 respondents with “early realization” had no idea of their children’s gender issues until their children disclosed their gender identities more than a decade later.

Turban et al. never explain why “disclosure” requires an explicit declaration of a (presumably fully formed) “TGD identity,” or why parents are incapable of picking up on relevant cues before such disclosure takes place. In their effort to push back against a straw man version of the ROGD hypothesis, Turban et al. go to the opposite extreme and assume that parental observations of their children’s behavior and stated desires are simply irrelevant.

## Time to Disclosure

The crux of Turban et al.’s argument is that what may appear as sudden realization and disclosure is, in most cases, actually a long-delayed disclosure of an earlier realized transgender identity. Turban et al.’s conclusion depends, as we have seen, on a number of questionable assumptions. In this section, we present the data from the ROGD-relevant age group even if those assumptions are accepted.

Table 1 presents the data and includes both early and late realization respondents. Using Turban et al.’s proxy for “realization” (Q3.1), the median time to disclosure in the “early realization” group is 11 years and the mode is 13 years. The mean time was 10.8 years (SD 4.1). However, in the “late realization” (ROGD presentation) group, both males and females had a mode of one year and a median of 3 years.^1^ The mean for males and females combined was 3.2 years (SD 2.8) (two sample *t*-test of difference between means of early and late realization, *t* = 79.6, *df* = 11,091, *p* < 0.001).

If we measure time to disclosure from starting to think one was trans (Q3.2), we find that in the ROGD-relevant age cohort, the “late realization” group has a mode and a median of one year, revealing that, for many youth, disclosure reflects a very recent adoption of a transgender identification..

**Table 1:** Time to disclosure (in years) for respondents aged 18–24, measured from Q3.1 (time feeling gender was different)

Subsample	Mode	Median	Mean	SD	*N*
“Early realization” (felt gender was different before age 11)					
Female	13	11	10.6	4.1	3,888
Male	11	12	11.3	4.2	1,324
“Late realization” (felt gender was different at age 11 or later)					
Female	1	3	3.1	2.6	4,436
Male	1	3	3.6	3.0	1,444

Statistics calculated using USTS-15 survey weights and omitting respondents who reported feeling a different gender after disclosing their trans identity

In short, the only age cohort in the USTS-15 that is relevant to ROGD analysis is the 18–24 age group. Within this group, answers from most respondents support the ROGD hypothesis. Turban et al. obscure this key finding by analyzing the entire USTS-15 sample, and within that sample only the early realization group. It is hard not to suspect that Turban et al. were actively avoiding analysis of the data that could support the ROGD hypotheses.

## Female Skew of Sample

Turban et al. report that 63.2% of the “later realization” group were “assigned female sex at birth,” another finding consistent with the findings in Littman’s (2018) original ROGD paper. Had Turban et al. narrowed their focus to the ROGD-relevant age group, they would have found that the female proportion in the USTS-15 was 75.2%. Turban et al. not only misrepresent the relevant data, they also fail to ask why, even if 62.3% of the sample is female, the sex ratio is skewed—and this, to recall, in a paper that purports to find evidence against a female-dominant phenomenon. The predominance of females also contradicts Turban et al.’s claims in another article that most transgender and gender diverse adolescents in the United States are “assigned male at birth” (Turban et al., 2022).

## Mental Health Profile of Respondents

Undisclosed by Turban et al. is the fact that the 18–24 year-old age group in USTS-15 reported significantly higher rates of psychological distress compared to older age cohorts (53 vs. 39% or lower). Although USTS-15 does not allow for causal inferences, the finding of high rates of distress is of interest considering that ROGD posits background mental health issues as a mechanism for developing gender dysphoria.

Turban et al. may believe that the high levels of comorbid mental health problems in young TGD people are due to “minority stress,” a theory borrowed from research on homosexuality (Hendricks & Testa 2012; Meyer, 2003; Testa et al., 2015) that has never been adequately tested in this context of TGD youth. Three considerations, however, make minority stress unlikely as the sole or even primary explanation for the higher rates of psychological distress in the USTS-15 18–24 year-old cohort. First, especially in the new adolescent-onset cohort associated with ROGD, mental health problems typically precede the onset of gender issues (Becerra-Culqui et al., 2018; Bechard et al., 2017; Kaltiala-Heino et al., 2015). One study found that gender dysphoric children are more likely than their peers to have experienced negative attachment patterns to caregivers and childhood trauma or loss (Kozlowska et al., 2021). ROGD hypothesizes that transgender identity may be a maladaptive coping mechanism for dealing with underlying mental health problems (Littman, 2018). Second, as confirmed in systematic reviews of the evidence, gender transition is not shown to cause mental health improvement (Brignardello-Peterson & Wierciacho, 2022; Ludvigsson et al., 2023; NICE, 2020a; 2020b; Pasternack et al., 2019). Third, there remains the possibility that gender dysphoria is inherently distressing, irrespective of the social context within which it arises.

Regardless of these considerations, if minority stress was the explanation, we would expect to see roughly similar levels of distress across age cohorts in USTS-15, or even lower levels among young adults given how young people tend to have more flexible views of sex and gender than Americans of older generations (Twenge, 2023). Instead, the older cohorts of TGD respondents to USTS-15 reported significantly lower levels of psychological distress.

These facts further support that many or most of the 18–24-year-old age group in USTS-15 fit the profile of ROGD. That Turban et al. never even considered this possibility demonstrates a lack of curiosity and scientific rigor.

## Inadequate Response to Critic

In a Letter to the Editor, Kulatunga-Moruzi (2023) argued that Turban et al. (2023a) relied on USTS-15 respondents' “self-reported memories,” which are “highly malleable and prone to distortions.” Turban et al. (2023b) dismissed the criticism, arguing that Littman’s original ROGD article contains a similar problem of “recall bias” (of parents rather than respondents) and adding that “[r]ecall bias is less of a concern when participants are being asked about major events in their own lives (e.g., coming to realize one’s TGD identity or sharing this with another person for the first time).”

We find Turban et al.’s response inadequate. The recovered memory movement is a case study in the suggestibility of memory surrounding sexual assault and abuse, obvious major life events (Loftus & Ketcham, 1994; Ofshe & Watters, 1994). The argument of “Age of Realization” relies entirely on the recalled memories of highly motivated survey respondents recruited through advocacy networks in a social context that encourages them to interpret their current gendered feelings as innate and immutable (“born that way”).

According to Turban et al.'s interpretation of USTS-15, 31% of respondents recalled realizing they were TGD by age 5. This includes 696 respondents (2.5%) who recalled realizing they were TGD by age 2 and 267 respondents (1%) who said that they knew this about themselves when they were just a year old. Turban et al. take these highly implausible recollections at face value.

Finally, considering their assumption that recollection of sexual identity development and change is infallible, we find it peculiar that Turban et al. ignored a recent study of detransitioners where respondents recalled social influence, mental health conditions, and internalized homophobia as factors influencing their decision to identify as TGD and undergo gender transition (Littman, 2021).

## Conclusion

The clinical presentation associated with ROGD has become internationally recognized (Bonfatto & Crasnow, 2018; Elkadi et al., 2023; Hutchinson et al., 2020; Zucker, 2019), with even the World Professional Association for Transgender Health now recognizing “the increased number of adolescents seeking care who have not seemingly experienced, expressed (or experienced and expressed) gender diversity during their childhood years” (Coleman et al., 2022). Turban et al. claim to find evidence against ROGD in USTS-15, but a more accurate analysis of that sample actually supports the ROGD hypotheses. Specifically, the USTS-15 data reveal that among younger respondents, ROGD presentation was common. For many respondents in the relevant age group, disclosure reflected a recent adoption of transgender identity. At a time when scientific debate over the phenomenon is badly needed, “Age of Realization” does more to obscure than to clarify the issues in this important debate.

## Footnotes

1 In response to criticism that they omitted data on time to disclosure in the late realization group (Kulatunga-Moruzi, 2023), Turban et al. reveal that the median time to disclosure in this group was 4 years. However, this figure applies to the entire sample, not to the ROGD age-relevant cohort (18–24 years).

## Declarations

### Editor's note

This Letter was peer-reviewed by two reviewers and by the Editor.
